# Relationship between research activity and the performance of English general practices: cross-sectional and longitudinal analyses

**DOI:** 10.3399/BJGP.2024.0111

**Published:** 2024-11-12

**Authors:** Jonathan Gibson, Evangelos Kontopantelis, Matthew Sutton, Annette Boaz, Paul Little, Christian Mallen, Richard McManus, Sophie Park, Juliet Usher-Smith, Peter Bower

**Affiliations:** National Institute for Health and Care Research (NIHR) School for Primary Care Research, University of Manchester, Manchester.; National Institute for Health and Care Research (NIHR) School for Primary Care Research, University of Manchester, Manchester.; National Institute for Health and Care Research (NIHR) School for Primary Care Research, University of Manchester, Manchester.; NIHR Health and Social Care Workforce Research Unit, King’s College London, London.; NIHR School for Primary Care Research, University of Southampton, Southampton.; NIHR School for Primary Care Research, Keele University, Keele.; Brighton and Sussex Medical School, Brighton.; NIHR School for Primary Care Research, University of Oxford, Oxford.; Department of Public Health and Primary Care, University of Cambridge, Cambridge.; National Institute for Health and Care Research (NIHR) School for Primary Care Research, University of Manchester, Manchester.

**Keywords:** family practice, general practice, outcomes, primary health care, research activity, research on research

## Abstract

**Background:**

Research activity usually improves outcomes by being translated into practice; however, there is developing evidence that research activity itself may improve the overall performance of healthcare organisations. Evidence that these relationships represent a causal impact of research activity is, however, less clear. Additionally, the bulk of the existing evidence relates to hospital settings, and it is not known if those relationships would also be found in general practice, where most patient contacts occur.

**Aim:**

To test 1) whether there are significant relationships between research activity in general practice and organisational performance; and 2) whether those relationships are plausibly causal.

**Design and setting:**

National data were analysed between 2008 and 2019, using cross-sectional and longitudinal analyses on general practices in England.

**Method:**

Cross-sectional, panel, and instrumental variable analyses were employed to explore relationships between research activity (including measures from the National Institute for Health and Care Research Clinical Research Network and the Royal College of General Practitioners) and practice performance (including clinical quality of care, patient-reported experience of care, prescribing quality, and hospital admissions).

**Results:**

In cross-sectional analyses, different measures of research activity were positively associated with several measures of practice performance, but most consistently with clinical quality of care and accident and emergency attendances. The associations were generally modest in magnitude; however, longitudinal analyses did not support a reliable causal relationship.

**Conclusion:**

Similar to findings from hospital settings, research activity in general practice is associated with practice performance. There is less evidence that research is causing those improvements, although this may reflect the limited level of research activity in most practices. No negative impacts were identified, suggesting that research activity is a potential marker of quality and something that high-quality practices can deliver alongside their core responsibilities.

## Introduction

Research is critical to improving quality of care and reducing variation in outcomes. England has a national research infrastructure (National Institute for Health and Care Research Clinical Research Network [NIHR CRN]),^1^^,^^2^ which has supported recruitment of several million patients, including crucial COVID-research platforms.^3^^–^5 There is a desire to further expand research participation to increase the amount and quality of research, reduce *‘research waste’*,^6^ and ensure that research is *‘conducted with and in the populations most affected’*.^7^

Research leads to impact when it generates benefits outside academia.^8^ In health and care settings a key benefit is implementation into practice, with much attention given to the gap between research evidence and routine practice.^9^ However, there are wider impacts of research, including developing evidence that participation in research by healthcare organisations may itself be related to better performance and improved patient outcomes, irrespective of the nature of the findings or whether they are subsequently implemented.^8^^–^10 For example, hospital participation in interventional studies in colorectal cancer is associated with improved survival among the wider patient population cared for by that hospital.^11^ Further studies and evidence syntheses have supported this hypothesis.^12^^–^14

However, evidence linking research activity and organisational performance largely comes from hospital settings, and similar benefits may not occur in general practice. General practices care for different patient populations, provide care that is less technical, and practices are smaller and more geographically distributed. Equally, the volume of research will be lower, types of research may be more varied, and only a proportion of the research activity may be focused on the priorities of general practice. There is an evidence base linking research activity in general practice to performance, but it is less extensive.^15^^–^17 Assessing the relationship in general practice is important, as the bulk of patient contacts are in this setting, and any benefits of research activity on general practice performance would be potentially widespread.

**Table:** How this fits in

There is developing evidence that undertaking research activity itself may improve the performance of healthcare organisations. However, the bulk of the evidence relates to hospital research, and it is less clear if these relationships represent a causal impact of research activity. This study showed that research activity in general practice is associated with a range of measures of practice performance. Research activity is a useful marker of high-performing general practices, but there is less evidence that research is causing improvements, possibly reflecting the limited levels of research activity in most practices.

Nonetheless, if these associations exist in both hospital and general practice settings, it cannot be assumed that research activity is causing better outcomes. Relationships between research activity and practice performance may be owing to other factors such as characteristics of practices or the patients they serve. As research activity is not amenable to experimentation, statistical modelling is required.

### Aims

This study sought to replicate existing evidence from hospital studies and 1) test whether there were significant relationships between research activity in general practice and organisational performance; and 2) assess whether those relationships were plausibly causal.

## Method

### Aim, design, and setting

The study aimed to assess whether levels of research activity in general practice were associated with the performance of general practices on a range of organisational and patient-reported outcomes. National data from general practice in England were analysed (between 2008 and 2019) using observational, panel, and instrumental variable models. Patients and the public advised on the analyses and interpretation.

### Measures of research activity

The NIHR CRN is divided into 15 local regions (https://local.nihr.ac.uk/lcrn) and provides national research activity data at a practice level on the following: 1) number of patients recruited by each general practice; and 2) the number of studies involving the practice. This measure was supplemented with a second measure provided by the Royal College of General Practitioners, as to whether practices were signed up to its Research Ready programme, which provides information and guidance to practices to support research activity. Practices were categorised as follows: 1) current members of the Research Ready programme; 2) previous members; or 3) practices that had never participated.

### Measures of practice performance

A logic model was developed with expert advisers and patient contributors to support the analyses that detailed measures, mechanisms, outcomes, and wider impacts on practice performance. A range of practice performance measures was used, based on national administrative and survey data, which captured several aspects of general practice performance and included more immediate impacts (such as patient experience) as well as those further down the causal pathway in the model (for example, hospital utilisation). The measures of practice performance were as follows:

- *Clinical quality of care*. Data were obtained from the Quality and Outcomes Framework (QOF) on points achieved in the clinical domains as a marker of the technical quality of care. As the number of points achievable changes annually, the percentage of points achieved in a particular year were used.
- *Prescribing quality.* The OpenPrescribing database was used to create a measure of the proportion of antibiotics issued that were narrow-spectrum antibiotics, which is a recognised marker of quality of general practice prescribing.^18^^,^^19^
- *Patient experience.* Data from the GP Patient Survey, which is independently administered and measures patient experience of general practice,^20^ were used on how responders 1) reported their overall experience with the practice, and 2) satisfaction with making an appointment. The percentage of patients who reported a ‘very good’ or ‘fairly good’ experience were analysed.
- *Hospital utilisation.* Counts of admissions (non-elective), outpatient attendances (first attendances and attended appointments only), accident and emergency (A&E) attendances, and ambulatory care sensitive conditions were obtained from Hospital Episode Statistics (HES) in 2017.
- *GP satisfaction and retention.* Data from the 2019 National GP Worklife Survey, which measures GP work–life experience, were obtained and linked to the practice. This could only be used in the cross-sectional analyses owing to differences in sampled GPs between years. From national workforce data, the percentage of GPs who remained at each practice from one year to the next was also calculated.^21^

The following covariates were also included: list size; full-time equivalent GPs, nurses, other direct patient care, and administrative staff; percentage of salaried GPs; local research network region; patient age and gender distribution; practice rural location; contract type; practice training status; market forces factor (a measure of wages in the local labour market); and income deprivation (in 2019). To construct the income deprivation score for a practice, the income deprivation proportions were summed for each of the practice’s patient-associated area. This sum was then divided by the total number of patients at the practice to get an average practice income deprivation score. A measure of population need was also included, based on the ratio of weighted to unweighted patients from the global sum allocation formula.^22^ The NHS uses weighted patients as a means of allocating ‘global sum’ payment to practices to account for workload. This weighting is based on a formula that includes patient need (morbidity and mortality). The ratio to unweighted patients was used as a measure of patient need. These covariates were obtained from published sources.^23^^–^26

### Statistical analyses

Cross-sectional analyses were initially used to explore relationships between cumulative research activity and practice outcomes. This was primarily to allow comparison with the wider literature using similar cross-sectional methods. Linear regression was used to relate practice performance to measures of research activity. For the cross-sectional analysis, the CRN data on numbers of patients and studies across the period were summed for which data were available (2008–2019 in some cases, with lesser periods with some analyses). The Research Ready measure is a binary indicator. Performance measures were standardised using z-score transformations to aid comparisons. The estimated effects of research were summarised by calculating a unit change in research (for example, an additional patient or study), holding other characteristics constant (median values for continuous variables, means for discrete variables). Huber–White robust standard errors were used to allow for heteroscedasticity.

The main analyses used panel models to explore relationships between annual research activity and practice performance in the following year. These analyses avoided reverse causality (as changes in research activity had to occur before practice outcomes) and controlled for unmeasured factors that are stable or relatively stable over time (such as practice research culture). The impact of research activity in a particular year on the outcome in the following year was examined using a fixed-effects regression model. The Research Ready measure did not vary over time and was excluded from the panel analyses.

The model is specified as follows:

$$Y_{it}=\beta_{1}X1_{it}+\ldots +\beta_{k}Xk_{it}+\beta Res_{it-1}+\alpha_{i}+u_{it}$$

in which *Y_it_* is the outcome for practice *i* in year *t, X1* to *Xk* are covariates, *Res_it-1_* is the research activity for practice *i* in year *t–1*, and α*_i_* are practice-specific intercepts that capture between-practice heterogeneity.

Panel models control for reverse causality and unmeasured factors that do not change over time but concerns about confounding remain if the practices that become research active also take other unmeasured actions at the same time to improve outcomes. To address this, an instrumental variables approach was used.^27^ An instrumental variable should be related to research activity (inclusion condition) and not otherwise impact on the outcomes directly (exclusion restriction). For our instrumental variables, measures of the amount of research activity in the local area were used (defined as the 15 local research networks covering England) as a predictor of the research opportunities available to the practice. A practice that is located in a high-activity area is potentially more likely to participate, relative to a practice located in a low-activity location, but wider research activity outside the practice is unlikely to impact on the performance of a specific general practice. To account for differences in the size of the local research area, the total activity was divided by the number of patients in the region. The following two measures were used to ensure that the instrumental variables model was over-identified: 1) the number of patients recruited into general practice research (per patient) in the local research network area; and 2) the same for secondary care research. It was assessed whether these instruments met conventional criteria (see Supplementary Appendix S1 and Table S1).

The reporting of the study conformed to the STrengthening the Reporting of OBservational studies in Epidemiology (STROBE) statement.

## Results

### Participating general practices

Research activity data were available for 7921 practices, of which 1465 (18.5%) were dropped owing to having no 2019 workforce data (indicating that they were no longer operating). A further 112 (1.7%) practices were excluded owing to list sizes <1000 (sub-practices, those attached to universities, and those closing down) and 141 (2.2%) were excluded as data on practice characteristics were unavailable. Descriptive statistics are presented in Table 1. Levels of research activity were generally low with high variation. Many practices scored highly on the outcomes used, especially clinical quality and overall satisfaction from patient-reported experiences of care.

**Table 1.** Descriptive statistics (research activity and outcomes)

**Variable**	**Obs**	**Mean**	**Standard deviation**	**Minimum**	**Maximum**
**Quality measures**					
QOF achievement (%)	6045	95.8	5.3	34.7	100
GP retention rate	5981	92.6	12.3	0	100
Patient satisfaction overall (%)	6061	83.8	9.6	39.5	100
Patient satisfaction access (%)	6061	69.6	14.5	19.1	100
Antibiotic ratio	6062	95.9	1.7	86.7	100
GP satisfaction	1045	4.6	1.6	1	7

**Recruitment activity**					
Patients recruited 2008–2019	6062	141.6	334.4	0	4602
Patients recruited 2015–2019	6062	67.3	207.9	0	3567
Studies 2008–2019	6062	6.9	9.5	0	92
Studies 2015–2019	6062	4.1	6.3	0	60

**Practice characteristics**					
Number of patients (000s)	6062	9.2	5.9	1.0	84.7
Patients: aged ≥65 years (%)	6062	17.8	6.9	0.01	49.4
Patients: female (%)	6062	49.9	2.1	19.4	61.1
GP FTE	6062	5.2	3.8	0.03	40.2
Nurse FTE	6062	2.5	2.3	0	32.1
Direct patient care FTE	6062	1.9	2.4	0	37.1
Administrative FTE	6062	10.3	7.8	0	106.7
GPs % salaried	6062	24.3	24.4	0	100
Years with trainees (0–8)	6062	3.1	3.2	0	8
Rural (1 = yes)	6062	0.17	0.38	0	1
GMS contract (1 = yes)	6062	0.72	0.45	0	1
Dispensing practice (1 = yes)	6062	0.17	0.37	0	1
Population need	6062	1.01	0.10	0.56	1.5
Market forces factor	6062	0.99	0.04	0.93	1.1
Income deprivation	6062	0.14	0.07	0.02	0.44

*FTE = full-time equivalent. GMS = General Medical Services. Obs = observations. QOF = Quality and Outcomes Framework.*

### Are there associations between research activity and organisational performance?

Cross-sectional associations between research activity and outcomes are shown in Table 2. The coefficients indicate the association between a unit change in research activity and a standard deviation change in the outcome. All measures of research activity showed a significant, positive association with clinical quality and a negative association with A&E attendances. The magnitude of these associations was small. For example, each additional research study (between 2008 and 2019) was associated with a 0.003 standard deviation increase in QOF achievement (*P*-value 0.006).

**Table 2.** Standardised cross-sectional regression models

	**Mean (SD)**	**Obs**	**Patients (100s) β (95% CI)**	***P*-value**	**Studies β (95% CI)**	***P*-value**	**Research Ready β (95% CI)**	***P*-value**
**Primary care outcome variables**								

QOF achievement (%)	95.8 (5.3)	6045	0.0079 (0.0020 to 0.0137)	0.0087	0.003 (0.001 to 0.006)	0.006	0.155 (0.061 to 0.249)	0.001
Antibiotic ratio	96.0 (1.7)	6062	−0.0043 (−0.0102 to 0.0016)	0.1503	−0.002 (−0.004 to 0.000)	0.081	−0.065 (−0.176 to 0.046)	0.248
Patient satisfaction overall (%)	83.8 (9.6)	6061	0.0050 (−0.0021 to 0.0121)	0.1703	0.005 (0.002 to 0.007)	<0.000	0.190 (0.088 to 0.291)	<0.000
Patient satisfaction access (%)	69.6 (14.5)	6061	0.0037 (−0.0034 to 0.0109)	0.3073	0.004 (0.002 to 0.007)	0.002	0.186 (0.069 to 0.304)	0.002
GP satisfaction	4.5 (1.6)	1045	0.0072 (−0.0066 to 0.0211)	0.3076	0.003 (−0.002 to 0.008)	0.274	−0.078 (−0.349 to 0.192)	0.570
GP retention rate	92.6 (12.3)	5981	0.0025 (−0.0043 to 0.0093)	0.4744	−0.001 (−0.003 to 0.002)	0.592	0.050 (−0.070 to 0.170)	0.414

**Secondary care outcome variables**								

ACSC (per 1000)	18.6 (5.6)	6080	−0.009 (−0.016 to −0.002)	0.008	−0.006 (−0.009 to −0.003)	<0.000	0.007 (−0.098 to 0.112)	0.893
A&E attendances (per 1000)	260.3 (83.8)	6080	−0.010 (−0.016 to −0.003)	0.003	−0.005 (−0.008 to −0.003)	<0.000	−0.106 (−0.193 to −0.020)	0.016
Emergency admissions (per 1000)	97.3 (25.4)	6080	−0.002 (−0.009 to 0.004)	0.504	−0.003 (−0.005 to −0.000)	0.029	−0.011 (−0.110 to 0.089)	0.836
Outpatient attendances (per 1000)	1579.3 (391.3)	6080	−0.008 (−0.015 to −0.001)	0.036	−0.005 (−0.008 to −0.002)	0.001	−0.032 (−0.146 to 0.083)	0.589

*A&E = accident and emergency. ACSC = ambulatory care sensitive conditions. Obs = observations. QOF = Quality and Outcomes Framework. SD = standard deviation.*

### Are associations between research activity and organisational performance causal?

The marginal effects for the fixed-effects panel models are shown in Table 3. Unlike the cross-sectional analyses, panel models showed far fewer significant relationships between research activity and practice performance in subsequent years. These were only in relation to research activity, as measured by number of research studies, and in different directions with different hospital outcomes.

The results of the instrumental variables analyses are shown in Table 4. There are few significant relationships between research activity and primary care outcomes. Post-estimation analysis of the instruments suggests they have reasonable power and validity, and there is little evidence of endogeneity bias (that is, unmeasured confounding; see Supplementary Table S1). This gives greater confidence that the results of the panel analyses of primary care outcomes are valid in showing no relationship between research activity and outcomes.

**Table 3.** Panel regression models

	**Mean (SD)**	**Obs**	**Patients β (95% CI)**	***P*-value**	**Studies β (95% CI)**	***P*-value**
**Primary care outcome variables**						
QOF achievement (%)	96.3 (5.9)	24 955	−0.001 (−0.011 to 0.010)	0.882	0.007 (−0.015 to 0.001)	0.076
Antibiotic ratio	95.8 (1.8)	25 158	0.002 (−0.010 to 0.015)	0.718	0.005 (−0.001 to 0.012)	0.111
Patient satisfaction overall (%)	84.8 (9.5)	25 104	0.003 (−0.011 to 0.016)	0.719	0.004 (−0.003 to 0.011)	0.284
Patient satisfaction access (%)	72.5 (14.2)	25 101	−0.004 (−0.016 to 0.008)	0.521	0.004 (−0.003 to 0.011)	0.245
GP retention rate	92.5 (13.2)	24 990	−0.007 (−0.027 to 0.014)	0.516	−0.004 (−0.015 to 0.008)	0.530

**Secondary care outcome variables**						
ACSC (per 1000)	18.5 (6.3)	12 876	−0.006 (−0.022 to 0.011)	0.494	0.012 (0.002 to 0.022)	0.020
A&E attendances (per 1000)	262.9 (96.5)	12 876	−0.003 (−0.017 to 0.012)	0.719	−0.008 (−0.014 to −0.001)	0.018
Emergency admissions (per 1000)	96.6 (31.8)	12 876	−0.009 (−0.023 to 0.005)	0.205	0.004 (−0.003 to 0.011)	0.262
Outpatient attendances (per 1000)	1593.8 (487.1)	12 876	0.000 (−0.005 to 0.006)	0.888	−0.004 (−0.009 to 0.001)	0.087

*A&E = accident and emergency. ACSC = ambulatory care sensitive conditions. Obs = observations. QOF = Quality and Outcomes Framework. SD = standard deviation.*

**Table 4.** Instrumental variable models

	**Mean (SD)**	**Obs**	**Patients IV β (95% CI)**	***P*-value**	**Studies IV β (95% CI)**	***P*-value**
**Primary care outcome variables**						
QOF achievement (%)	96.28 (5.86)	24 955	−0.045 (0.234 to 0.144)	0.642	−0.015 (−0.095 to 0.065)	0.707
Antibiotic ratio	95.80 (1.76)	25 158	0.126 (0.034 to 0.285)	0.123	0.062 (−0.011 to 0.135)	0.095
Patient satisfaction overall (%)	84.77 (9.49)	25 104	0.183 (0.014 to 0.351)	0.034	0.017 (−0.046 to 0.081)	0.592
Patient satisfaction access (%)	72.52 (14.18)	25 101	0.083 (0.063 to 0.229)	0.263	0.074 (−0.010 to 0.158)	0.083
GP retention rate	92.47 (13.23)	24 990	0.277 (0.036 to 0.589)	0.083	0.109 (−0.025 to 0.242)	0.111

**Secondary care outcome variables**						
ACSC (per 1000)	18.52 (6.26)	12 876	−0.086 (0.297 to 0.125)	0.424	−0.062 (−0.198 to 0.074)	0.375
A&E attendances (per 1000)	262.89 (96.49)	12 876	−0.371 (−0.558 to −0.183)	<0.000	−0.280 (−0.404 to −0.155)	<0.000
Emergency admissions (per 1000)	96.57 (31.75)	12 876	0.160 (0.001 to 0.320)	0.049	0.040 (−0.055 to 0.134)	0.414
Outpatient attendances (per 1000)	1593.82 (487.06)	12 876	−0.320 (−0.461 to −0.178)	<0.000	−0.179 (−0.261 to −0.097)	<0.000

*A&E = accident and emergency. ACSC = ambulatory care sensitive conditions. Obs = observations. QOF = Quality and Outcomes Framework. SD = standard deviation.*

In terms of secondary care outcomes (Table 4), instrumental variable analyses show more significant associations between research activity and outcomes than the panel analyses. Post-estimation analysis shows the instrumental variables have reasonable power and validity, and that some of the panel analyses may be subject to unmeasured confounding. However, in these cases where the instrumental variable analysis may be adding value, the directions of effect are inconsistent (for example, showing that increased patient recruitment leads to increases in emergency department use and decreases in outpatient attendance).

## Discussion

### Summary

National longitudinal data on research activity and general practice performance were used to demonstrate that, in line with the wider hospital literature, research activity was associated with practice performance, including quality of clinical care, patient experience, and hospital utilisation. However, further analyses did not provide supportive evidence that these relationships were causal, with little consistent evidence (either in terms of direction or statistical significance) showing effects of past research activity on improved practice performance in the longitudinal analysis. Therefore, across the set of analyses conducted, results do not support a strong message about causal impacts of research activity.

### Strengths and limitations

The analyses were comprehensive in terms of the population of practices and access to data on their characteristics. Nevertheless, there were limitations. Practices may engage in research activity not captured by NIHR CRN, including identification of patients for hospital studies where there is no consent in primary care. Others engage in database projects, such as the Clinical Practice Research Datalink or ORCHID, which may involve data quality initiatives, but where the involvement of practitioners may be less than studies that involve more active research processes (such as patient identification and consent) or interventional research, involving the delivery of new treatments to patients or the introduction of new care pathways. There may be wider activities, such as audit and service evaluation, which are not formally captured as research but may involve similar processes and may be important markers of better care. Although the authors had access to a number of measures of performance, which have been widely used in other research, these were routine measures, were not chosen on the basis of links to the research undertaken, and may have features (such as low variation around generally high-performance levels as in the QOF indicators)^28^ that make them less discriminating as measures of quality. There will be a direct contribution of research activity in individual practices to the combined research activity in the area, which will generate a modest degree of endogeneity in the instrumental variable. In addition, regional organisations may undertake quality improvement activities as well as encouraging research participation. This would invalidate the instruments but is unlikely given the disparate organisations involved. Finally, the analyses pre-dated the pandemic and may not capture benefits of large-scale engagement in COVID-19 studies.

### Comparison with existing literature

The present study is one of the largest assessments of the link between research activity and performance, compared with previous studies in general practice.^15^^–^17 A recent study using similar data to the present study has replicated the cross-sectional associations reported here for patient satisfaction.^29^ The present study adds value as it included a wider range of outcomes and measures of research activity, and importantly moved beyond cross-sectional analyses to explore causal relationships.

As noted previously, the lack of effects in the panel analyses may not reflect the general practice context specifically, as most analyses in hospitals are cross-sectional.^12^ Nevertheless, there are features of general practice that might attenuate impacts of research activity. First, the ‘dose’ of research activity in general practice is low: the mean annual patients recruited in 2015–2019 was 11. In a highly cited article linking hospital research to outcomes,^11^ hospitals showing the biggest impacts on outcomes reported 25% of patients with colorectal cancer in studies, a qualitatively different level of activity. Additionally, the hospital study had a very focused scope, involved a single specialty with a high number of clinical trials, which had the aim of changing clinical practice and impacting on a defined outcome (mortality), the optimal conditions in which to find strong relationships. In contrast, general practice research may involve a far wider range of studies on diverse topics that do not map neatly onto the measures of general practice performance available for the present study (for example, top-recruiting general practice research 2018–2019 included studies of improved check-in facilities, vascular genetics, and diagnostic testing in Barrett’s oesophagus). Only a minority of studies in general practice would be specifically related to the present study’s outcomes (for example, prescribing). The authors did not have detailed data on the research studies that would have supported subgroup analyses, such as linking antibiotic research activity with the prescribing outcomes, or research on quality of care with the QOF outcomes.

### Implications for research and practice

There is interest in increasing research activity to better serve the NHS needs, and the idea that such increases would also lead to ‘spill-over’ benefits in practices is an attractive one. The results suggest that research activity remains a useful indicator of a high-performing general practice. Importantly, patient contributors involved in the study raised some concerns that general practice research could distract from clinical responsibilities. However, no evidence was found that research activity was associated with any consistent reductions in performance (such as patient experience of access).

Levels of research activity in general practice are relatively low and highly variable, and the case for greater investment in primary care research remains strong.^30^^,^^31^ It is possible that higher levels of research activity are associated with more significant impacts. This may be more likely if research activity is augmented with additional facilitation that could maximise spill-over benefits (such as providing practices with more feedback, or more time to reflect on the implications of research), or if research activity involves types of research that may be better able to generate wider benefits.^15^

Increasing research activity may not be a reliable way of improving general practice performance. Nevertheless, research activity is a useful indicator of a high-performing practice and is not associated with any consistent reductions in measures of practice performance.

## Data

Raw research activity and outcome data can be requested from the appropriate organisations that hold that data, except where publicly available.
